# Efficacy and safety of supplemental melatonin for delayed sleep–wake phase disorder in children: an overview

**DOI:** 10.1016/j.sleepx.2020.100022

**Published:** 2020-08-19

**Authors:** David Mantle, Marcel Smits, Myrthe Boss, Irene Miedema, Inge van Geijlswijk

**Affiliations:** a Pharma Nord, Morpeth, UK; b Multidisciplinary Expertise Centre for Sleep-Wake Disorders and Chronobiology, Gelderse Valley Hospital Ede, The Netherlands; c Utrecht Institute for Pharmaceutical Sciences (UIPS), Department of Pharmacoepidemiology and Clinical Pharmacology, Faculty of Science, and Faculty of Veterinary Medicine, Pharmacy Department Utrecht University, Utrecht, The Netherlands

**Keywords:** Melatonin, Children, Adolescents, Delayed sleep–wake phase disorder, DSPD, Safety

## Abstract

Delayed sleep–wake phase disorder (DSPD) is the most frequently occurring intrinsic circadian rhythm sleep–wake disorder, with the highest prevalence in adolescence. Melatonin is the first-choice drug treatment. However, to date melatonin (in a controlled-release formulation) is only authorised for the treatment of insomnia in children with autism or Smiths-Magenis syndrome. Concerns have been raised with respect to the safety and efficacy of melatonin for more general use in children, as melatonin has not undergone the formal safety testing required for a new drug, especially long-term safety in children. Melatonin is known to have profound effects on the reproductive systems of rodents, sheep and primates, as well as effects on the cardiovascular, immune and metabolic systems.

The objective of the present article was therefore to establish the efficacy and safety of exogenous melatonin for use in children with DSPD, based on in vitro, animal model and clinical studies by reviewing the relevant literature in the Medline database using PubMed.

Acute toxicity studies in rats and mice showed toxic effects only at extremely high melatonin doses (>400 mg/kg), some tens of thousands of times more than the recommended dose of 3–6 mg in a person weighing 70 kg. Longer-term administration of melatonin improved the general health and survival of ageing rats or mice. A full range of in vitro/in vivo genotoxicity tests consistently found no evidence that melatonin is genotoxic. Similarly long term administration of melatonin in rats or mice did not have carcinogenic effects, or negative effects on cardiovascular, endocrine and reproductive systems.

With regard to clinical studies, in 19 randomised controlled trials comprising 841 children and adolescents with DSPD, melatonin treatment (usually of 4 weeks duration) consistently improved sleep latency by 22–60 min, without any serious adverse effects. Similarly, 17 randomised controlled trials, comprising 1374 children and adolescents, supplementing melatonin for indications other than DSPD, reported no relevant adverse effects. In addition, 4 long-term safety studies (1.0–10.8 yr) supplementing exogenous melatonin found no substantial deviation of the development of children with respect to sleep quality, puberty development and mental health scores. Finally, post-marketing data for an immediate-release melatonin formulation (Bio-melatonin), used in the UK since 2008 as an unlicensed medicine for sleep disturbance in children, recorded no adverse events to date on sales of approximately 600,000 packs, equivalent to some 35 million individual 3 mg tablet doses (MHRA yellow card adverse event recording scheme).

In conclusion, evidence has been provided that melatonin is an efficacious and safe chronobiotic drug for the treatment of DSPD in children, provided that it is administered at the correct time (3–5 h before endogenous melatonin starts to rise in dim light (DLMO)), and in the correct (minimal effective) dose. As the status of circadian rhythmicity may change during long-time treatment, it is recommended to stop melatonin treatment at least once a year (preferably during the summer holidays).

## Highlights

• Melatonin improves sleep onset without serious adverse effects in youths with DSPD. Change th text after the fourth bullet into: Melatonin is an efficacious and safe chronobiotic drug for the treatment of DSPD in youths.
• Melatonin for indications other than DSPD, dose not cause relevant adverse effects.
• Long term melatonin treatment does not impair sleep, puberty, and mental health.
• Melatonin is an efficacious and safe chronobiotic drug for the treatment of DSPD in youths.
• Melatonin should be administered at the correct time and in the minimal effective dose.

## 1 Introduction

Delayed sleep–wake phase disorder (DSPD) is the most frequently occurring intrinsic circadian rhythm sleep–wake disorder [1], with the highest prevalence in adolescence [2]. DSPD is characterized by difficulty in falling asleep and waking in the morning, while sleep duration and quality are usually normal DSPD can affect children both with and without associated mental or neurological problems. In DSPD the endogenous melatonin rhythm is delayed, and is no longer in alignment with the desired sleep time. This misalignment explains the difficulty falling asleep and waking in the morning, and may result in daytime sleepiness, poor school performance, anxiety, and behavioural problems [3].

There is convincing evidence for the short-term efficacy of non-medical chronobiological treatments for DSPD, including behavioural and light therapy. However, long-term treatment outcomes can be improved [4,5]. For those subjects for whom non-pharmacological treatments have been unsuccessful, melatonin is considered as first-choice drug treatment for DSPD [6].

Exogenous melatonin is a chronobiotic drug with some soporific and hypnotic properties [6,7]. It may shift circadian rhythms, including the sleep–wake cycle and is increasingly used to improve sleep in children and adolescents [6]. However, melatonin is not authorised for general use in children [8]; melatonin (in controlled-release form) is currently authorised for use in children with autism or Smiths-Magenis syndrome in European Community countries, but not for more common sleep disorders such as DSPD. Supplemental (exogenous) melatonin is available in both controlled-release and immediate-release formulations. For sleep induction, as is required for DSPD, immediate-release melatonin is considered to be the more effective formulation [9], and this is the melatonin form used in most clinical studies on DSPD. Food grade melatonin is available over-the-counter (OTC) in some countries. The manufacturing quality and bio-availability of melatonin differs in these unlicensed melatonin preparations [10]. Consequently there is a clinical need for an authorised immediate-release melatonin medicinal product.

Although there is a clear rationale for the use of supplementary melatonin in DSPD in children, concerns have been raised with respect to safety and efficacy. Melatonin has not undergone the formal safety testing expected for a new drug, especially long-term safety in children; it is known to have profound effects on the reproductive systems of rodents, sheep, and primates, as well as effects on the cardiovascular, immune, and metabolic systems. In addition, there is the potential for important interactions with drugs sometimes prescribed for children [[11], [12], [13]]. Furthermore, the efficacy of melatonin for insomnia has been questioned, although in part this can be ascribed to the heterogeneity of the sleep disorders studied, varying from largely unspecified insomnia to the better defined DSPD [14],as well as co-morbidities associated with the described sleep disorder [15,16].

The purpose of this paper is therefore to review evidence from the literature relating to safety and efficacy of exogenous melatonin for the treatment DSPD in children.

## 2 Methods

The published literature in the Medline database was searched via PubMed for relevant articles on the efficacy and safety of exogenous melatonin using in vitro, animal model and clinical studies, the latter specifically via randomised controlled trials in children.

## 3 Delayed sleep–wake phase disorder

DSPD is a circadian rhythm sleep disorder, first described by Weizmann et al., in 1981 [17], and characterized by habitual sleep–wake times that are delayed in comparison to conventional or socially acceptable sleep times [18]. When allowed to choose their preferred sleep–wake schedule, individuals with DSPD will exhibit normal sleep quality and duration for age [19], although with deviated timing.

DSPD is the most frequent circadian rhythm sleep disorder [20] and is particularly common among adolescents, with a prevalence of 7–16% [21,22], compared to a prevalence in adults of 0.13–0.17% [23,24].

Patients with DSPD complain of difficulty in falling sleep, and not obtaining sufficient sleep on school or work nights. Such individuals often have difficulty rising at a socially acceptable wake time. DSPD is associated with poor school adherence, lower school grades, smoking, alcohol usage, anxiety, and depression [[25], [26], [27], [28]].

Several co-morbidities are associated with DSPD, including attention-deficit/hyperactivity disorder (ADHD) and autism spectrum disorder (ASD) [6,[29], [30], [31], [32], [33]]. Co-morbidity and social features may mask characteristic symptoms of DSPD. Thus patients, especially children with DSPD and ADHD or ASD, may have less trouble waking in the morning than DSPD patients without these co-morbidities. Consequently chronic sleep onset insomnia can be the single mean characteristic. In children, sleep onset insomnia is sometimes considered as an issue for the caregiver and not necessarily for the child. However, in DSPD the sleep disturbance is associated with impairment of social, occupational or other areas of functioning [2] and consequently is an issue also for the child.

In DSPD endogenous circadian rhythms are not in accordance with socially conventional and desired times for sleep/wake. Several genetic, physiological and behavioural mechanisms have been suggested but there is sparse evidence for most of these [34]. An abnormally long intrinsic circadian period τ is crucial in the pathogenesis of DSPD [35]. A length polymorphism in the PER3 gene was reported to be linked to DSPD and extreme diurnal preference [36]. Up to 40% of those affected by DSPD may have a family history of this disorder, possibly associated with this polymorphism [37].

In mammals, the temporal organization of metabolism, physiology, and behaviour around 24 h is controlled by a network of multiple cellular clocks, synchronized via neuronal and hormonal signals by a master clock located in the suprachiasmatic nuclei (SCN), and peripheral clocks located throughout the body. The SCN clock is set to solar time by photic input pathways originating in the retina, while secondary circadian clocks in other brain areas and peripheral clocks can be reset by meal timing [38,39]. This explains why irregular meals are adversely associated with metabolic risk [40].

Alterations in entrainment of the circadian clock to synchronizing agents such as light and physical activity contribute to the delayed timing of sleep [25]. Individuals with DSPD may have an altered responsiveness to light [41], and are more sensitive to evening light [42]. Furthermore, alterations in the homeostatic regulation of sleep and inappropriate meal timing (chrononutrition) may also play a crucial role in the development and maintenance of DSPD [43,44].

Current treatments for DSPD include light therapy, behavioural, and environmental approaches [4,[45], [46], [47]]. There is convincing evidence for the short-term efficacy of these chronobiological treatments for DSPD. However, relapse of symptomology is common [4], and long-term treatment outcomes can be improved [4,5]. Therefore supplemental melatonin is often recommended [6].

### 3.1 Rationale for the use of exogenous (supplemental) melatonin to enhance sleep

**Fig. 1:**
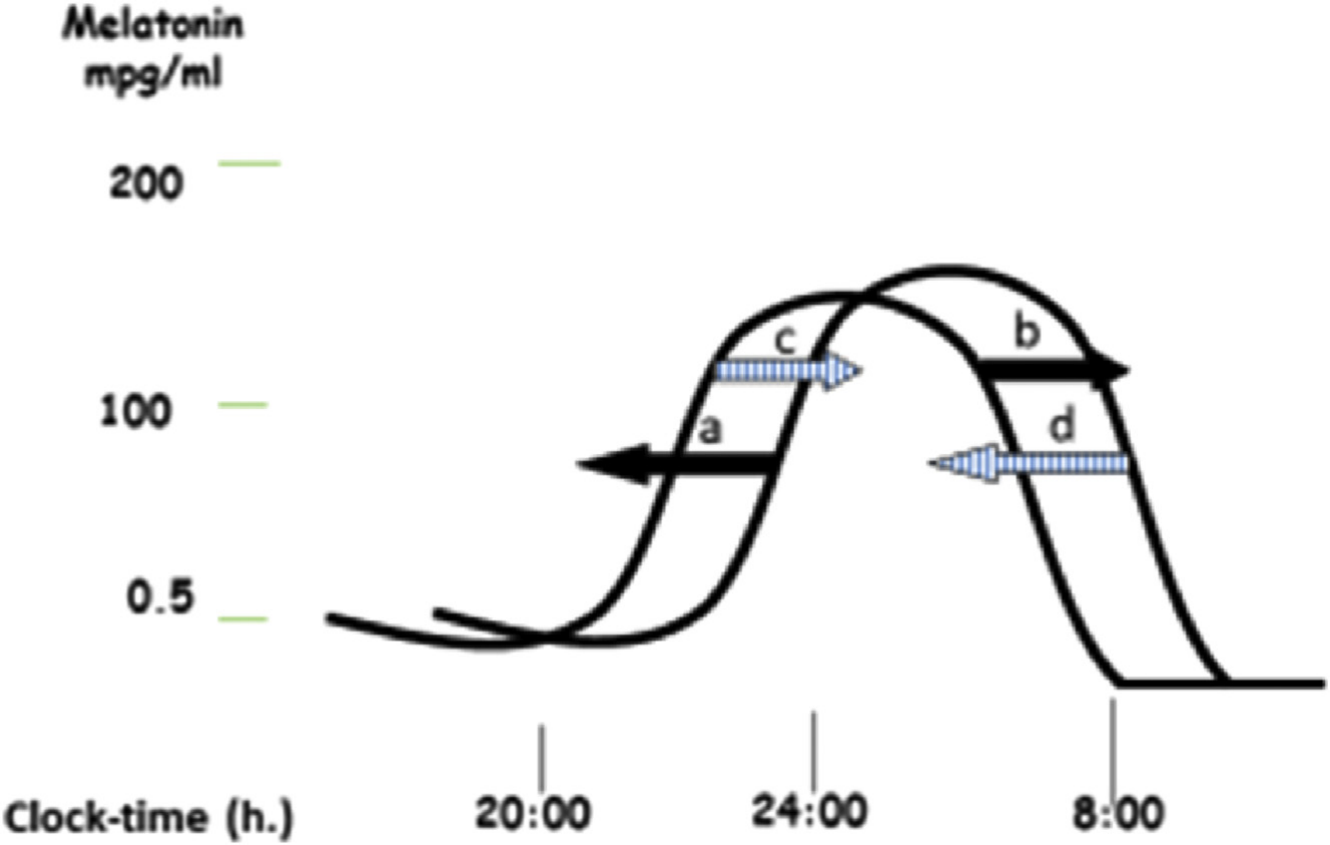
Exogenous-melatonin-induced shifts and bright-light-induced shifts of the endogenous melatonin rhythm. The endogenous melatonin rhythm is advanced the most when exogenous melatonin is administered 5 h before the dim light melatonin onset (DLMO) (a); when administered 10 h after DLMO it is delayed the most (b). Bright light during the increasing phase of the melatonin curve delays the endogenous melatonin rhythm (c). Bright light during the decreasing phase of the melatonin curve advances the endogenous melatonin rhythm (d). As the natural sleep–wake rhythm is associated with the endogenous melatonin rhythm, exogenous melatonin- and bright light-induced shifts of the endogenous melatonin rhythm parallel sleep–wake rhythm shifts.

Exogenous (supplemental) melatonin influences sleep mainly by its interaction with endogenous melatonin. Therefore the influence of endogenous melatonin on sleep is summarized, followed by a discussion of the most powerful influencers on endogenous melatonin ie exogenous melatonin and bright light (summarised in Fig. 1).

#### 3.1.1 Relationship between endogenous melatonin and sleep

To achieve consolidated sleep (and wakefulness), two processes should interact and balance each other. The first includes a homeostatic sleep drive (process S), determined by recent sleep–wake history such that the longer you are awake, the more the homeostatic pressure to sleep. The second is the circadian rhythm (process C), which is largely independent of recent sleep and waking activity. It influences the timing, duration, and internal structure of sleep, and is regulated by a central endogenous circadian pacemaker (ECP) located in the suprachiasmatic nuclei (SCN) of the hypothalamus. The ECP regulates many biological functions, such as sleep, metabolism, and temperature. The ECP has a slightly-more-than-24-h period which is synchronized to geophysical time by regular exposure to light and darkness [48].

The ECP has a higher frequency of firing during the day compared to the night. In reaction to a decrease in firing of the ECP during the night, melatonin is secreted by the pineal gland. The timing of its secretion is influenced by several clock-genes (eg PER3, PER2, Clock) [49]. Furthermore, melatonin secretion is suppressed by light [50], particularly blue light [51]. Physiological melatonin levels begin to rise in the evening, peak during night-time and decrease early in the morning: they are low or absent during the daytime (Fig. 1).

Melatonin exerts its physiological action via MT1 and MT2 receptors, located particularly in the SCN, but also in other areas of the brain. Melatonin inhibits SCN neuronal firing via the MT1 receptors, and activation of MT2 receptors mediates melatonin's phase shifting effects, although a role of the MT1 receptor on phase shifting cannot be excluded [51,52]. Melatonin may also alter the functions of the GABA-A-benzodiazepine receptor complex. These effects, linked to the activation of GABAergic mechanisms in the SCN, are putative mechanisms by which melatonin mediates the circadian timing of the sleep–wake cycle [[53], [54], [55]].

The time at which melatonin starts to rise in dim light, the so-called Dim Light Melatonin Onset (DLMO) is the characteristic of the 24-h melatonin curve which is most relevant for clinical practice [56]. When endogenous melatonin starts to rise (usually late in the evening), the possibility to fall asleep increases [57]. When other circumstances are also favourable (eg enough rest, lying in bed, absence of mental activities, etc.), one may fall asleep, and wake up rested some 8–9 h later. When endogenous melatonin starts to rise later than at a conventional time, sleep-onset and sleep offset are delayed. Patients then suffer from sleep-onset insomnia and have difficulty waking up in the morning at a conventional time. Thus, the discrepancy between biological and social clocks, so-called “social jetlag”, occurs [58,59]. When melatonin starts to rise earlier than at a conventional time, the sleep–wake rhythm is advanced. Thus patients fall asleep too early in the evening and wake up too early in the morning.

The 24-h endogenous melatonin rhythm is associated with the 24-h temperature rhythm. This association is in an opposing direction: when melatonin increases, temperature decreases and vice versa. Circadian variations in temperature may act as an input signal to sleep-regulating systems [60].

The slightly-more-than-24-h circadian rhythm is corrected to a 24-h rhythm by bright light in the morning. Consequently the sleep–wake rhythm in most people follows a 24-h rhythm. Failure of bright light exposure in the morning results in an automatically occurring delay of the sleep–wake rhythm. This natural synchroniser of the endogenous melatonin rhythm also explains why people adapt more easily to changing time zones after westward travel rather than after eastward travel. Thus eastward travel is paralleled by automatically occurring delayed sleep–wake rhythm, which needs to be advanced. Bright light in the morning, especially during the phase when endogenous melatonin is decreasing, or exogenous melatonin, administered 5 h before DLMO (as mentioned in Fig. 1, to be explained later in this review) will help to advance the endogenous melatonin rhythm and the sleep–wake rhythm that is associated with it [61].

The natural tendency to delayed sleep–wake rhythms seems to be stronger during adolescence than in other phases of life [58], mostly due to an age related physiological process. Additional to this natural tendency, adolescents tend to use electronic devices more frequently than adults for social media during the rising phase of the melatonin curve, which might enhance the delay of the sleep–wake rhythm (Fig. 1). Due to these two factors, advancing the delayed sleep–wake rhythms of adolescents requires much more effort than in adults. This might be a major reason for the increased prevalence of DSPD in adolescents when compared to adults, although new technologies in cell phones and other electronic devices reduce this exposure in blue light, and eventually only the evolutionary difference between adolescents and adults will remain.

### 3.2 Influence of exogenous melatonin and bright light on endogenous melatonin and sleep

Exogenous melatonin may shift the endogenous melatonin rhythm, and with it the associated circadian rhythms, including the sleep–wake rhythm. The direction of the shift depends upon the time at which exogenous melatonin is administered. The melatonin (and the sleep–wake) rhythm is advanced most when in adults exogenous melatonin is administered 5 h before DLMO. Consequently it is an effective treatment for circadian rhythm sleep disorders, especially DSPD [62]. In clinical practice, melatonin is administered for (adolescent) DSPD patients not earlier than 19:00 h, otherwise the hypnotic effects of melatonin induce sleepiness too early in the evening. Exogenous melatonin delays endogenous melatonin rhythm (and the sleep–wake rhythm) most when administered 10 h after DLMO [63]. Consequently it can be used to delay sleep–wake rhythm in advanced sleep phase disorder (ASPD). Melatonin should then be taken somewhere in the middle of the night. However, in clinical practice ASPD is preferably treated with bright light early in the evening, during the rising phase of melatonin production.

Several pharmacopeias, and also the European Food Safety Authority, as well as all summaries of product characteristics (SPCs) of authorized medicinal products advise to take melatonin at a time (eg half an hour) before (desired) bed time [64]. However, as discussed above this is incorrect; the correct time to administer melatonin is related to the DLMO and not the desired bedtime. This was confirmed in children with chronic sleep-onset insomnia and late DLMO, showing a significant correlation between DLMO-determined time of administration and treatment effect on DLMO and sleep-onset time [65]. As the DLMO cannot be estimated in patients with sleep disorders [66], DLMO has to be measured. Fortunately, this can easily be done by collecting saliva at the patients home [48]. When melatonin administration does not have the desired effect within 1–2 weeks, administration time and/or dose should than be modified, as described below.

Not only the administration time, but also the dosage, is important for correct melatonin treatment. When the dose of melatonin administered in the evening is too high, the next day not all exogenous melatonin is metabolised, and melatonin remains present in the body. When the following evening melatonin is administered again in a too high dose, the melatonin in the body will increase again (accumulation). This spill-over will cover up the endogenous melatonin rhythm and the exogenous melatonin loses its efficacy. Accumulation may occur in “normal” doses when melatonin is metabolised slowly [67]. This slow melatonin metabolisation is associated with polymorphisms of the CYP1A2 gene, which occurs in 5–10% of the population, and possibly more in patients with autism spectrum disorder [68]. In that case the melatonin dose should be lowered, eg to 0.1–0.3 mg. For optimal treatment effects melatonin should be timed and dosed individually. Consequently melatonin treatment is an example of personalized/precision sleep medicine [66].

As noted above, light also may shift endogenous melatonin rhythm. Therefore light therapy is also recommended for the treatment of circadian rhythm sleep disorders [62], possibly in combination with melatonin treatment [69]. However, there is not enough evidence for the efficacy of the former [70]. A study comparing bright light therapy with melatonin treatment in children with DSPD showed only slight effects of bright light, while the effects of melatonin were much stronger [71].

## 4 Pharmacokinetics of melatonin

Melatonin is present in multiple tissues within the body. Due to its small molecular size and lipophilic nature, melatonin moves rapidly across cell membranes. Most circulatory melatonin is reversibly bound to albumin, is distributed to all tissues, and can cross the blood–brain barrier. Melatonin is principally metabolised in the liver; the elimination half-life is 35–45 min in extensive metabolizers [72], but might increase to 6.6 h in poor metabolizers [68]. The level of endogenous melatonin production is fixed after age 1 yr, probably because the pineal gland does not appear to grow, in contrast to the pituitary gland that doubles in size. This explains why endogenous melatonin is high in early childhood, and decreases in relation to body size [73]. Exogenous melatonin is efficiently absorbed from the digestive tract, but the bioavailability is low (ie <15%) due to first pass elimination [74]. This first pass effect can be by-passed by oro-mucosal (buccal) administration. The rate of clearance in children is greater than in adults, because in children enzymatic activity of CYP1A2 (the principal metabolizer of melatonin) in the liver is increased [75]. Children require a relative-to-bodyweight higher dose to induce sleep than adults. Most clinical studies in children with DSPD have been performed with immediate-release melatonin preparations.

In order to mimic the endogenous melatonin profile, a controlled-release melatonin preparation was developed. A paper by Chua et al. published in 2016 in the journal Pharmaceutics recommended the use of divided or crushed Circadin tablets (as a licensed product) where an immediate release melatonin was required, in preference to a manufactured immediate release tablet (unlicensed in the UK) [76]. However what the authors had not stated was that this represents off-license use of a licensed medicine, promotion of which is illegal [77]. Clinical studies to compare the efficacy of immediate- and controlled-release melatonin preparations have not yet been published [78].

## 5 Melatonin supplement formulation

Supplemental (exogenous) melatonin is available in three different formulations, immediate-release orally and oro-mucosally, and oral controlled-release. Immediate-release oral melatonin is worldwide the most frequently used formulation, often as an over-the-counter (OTC) product. There are no melatonin-based medicinal products authorised for use in DSPD, or for generalised use in children, but medicinal products authorised for adults with jetlag are available in some EU countries. The active content of unlicensed OTC melatonin products, and potential contaminants, can vary widely [10,79]. For sleep induction (as is required for DSPD), immediate-release melatonin is considered more effective, whilst controlled-release melatonin is considered to be more effective for sleep maintenance. Controlled-release melatonin was developed to mimic the nocturnal release of endogenous melatonin, for example for the treatment of certain types of insomnia in the elderly with pineal gland deficiency [79,80]. However, since there is no strong evidence that the amount of melatonin produced at night directly influences sleep quality [45], there seems to be no reason to prefer controlled-release above immediate-release formulations in children. Immediate-release formulations typically consist of the active melatonin dose dispersed in an inactive (usually cellulose-based) matrix. Taking Bio-Melatonin as an example, complete tablet disintegration occurs within 15 min in the pH range 1.2–6 [77], well within the ICH (International Conference on Harmonisation) guideline requirements for immediate-release tablets (80% tablet dissolution at 30 min). In the case of controlled-release formulations, the matrix serves to control the release of the melatonin activity over a more sustained time period. It is of note that controlled-release formulations may release up to 25% of the total dosage on an immediate basis [81], so there is a degree of overlap between the formulations.

In addition to controlled-release formulations of melatonin, melatoninergic drugs with a longer half-life have been developed, such as ramelteon, tasimelteon, and agomelatine, for (seasonal) depression. With all these medicinal products, improvements of sleep are statistically demonstrable, but clinically relevant effects remain limited [82].

## 6 Clinical efficacy studies with melatonin in children

### 6.1 Delayed sleep phase disorder

A total of 19 randomised, double blind, placebo-controlled trials assessed the efficacy of melatonin in more than 800 children and adolescents with DSPD phenotypes [77]. The most frequently used dosage was 3–6 mg/day. Eighteen studies used an immediate-release melatonin formulation, and one study used a mixed immediate-release/controlled-release formulation. Time of administration varied between approximately 30 min prior to bedtime and 2–3 h before DLMO. Most studies reported an improvement in sleep latency of at least 30 min. A meta-analysis of 7 randomized controlled trials assessing efficacy and safety of melatonin in 387 children and adolescents concluded that melatonin advanced mean sleep-onset time by 37 min and DLMO by 49 min [83]. Sleep was measured using sleep-dairies in all studies, and actigraphy in 5 studies. In four of the latter studies [14,[84], [85], [86]], sleep-times before and after melatonin treatment were described, making comparison with published normal values possible [87]. Mean sleep latency decreased significantly from 52–63 min to 27–36 min (normal value: 19 min), and mean sleep-onset times in from 21:40–22:45 h to 21:00–21:42 h.

#### 6.1.1 Melatonin dose

Melatonin does not show a dose–response relationship within a dose range of 0.05–0.15 mg/kg with regard to sleep-onset time, sleep-onset latency and DLMO [65]. This suggests that melatonin activates a “switch” in the brain, resulting in a shift of circadian rhythmicity. For clinical use this means that it is pointless to increase the dose further, when melatonin has been effective. Probably most important for efficacy is the time of administration (TOA) in relation to the DLMO, and in extensive metabolizers the highest dose might seem more effective when this interval of TOA and DLMO is increased (ie the dose is taken earlier, or the DLMO is severely delayed), while a lower dose might be as efficacious when the interval is shorter [65].

#### 6.1.2 Duration of melatonin treatment

In most placebo-controlled studies the duration of melatonin treatment was 4 weeks. Some studies reported results of treatment continuous up to 10.8 years, showing that after 3.1 years 20% of the children successfully stopped melatonin treatment [88,89], and after 10.8 years this figure increased to 75%. The sleep disturbance might simply be better tolerated by the individual or by the carers as the person becomes older, enabling the melatonin to be “successfully stopped”.

The differences in characteristics of the children who were able to stop, and who were unable to stop melatonin supplementation are unknown. To establish if treatment is still necessary it is advisable to stop melatonin treatment every year, preferably for a few weeks during the summer holidays; stopping treatment then usually influences quality of life as little as possible.

### 6.2 Other disorders

The efficacy of melatonin has been studied in randomised controlled trials of children in situations other than DSPD. These include studies on migraine [90], Dravet syndrome [91], atopic dermatitis [92], medication induced weight gain in bipolar disorder [93], and as sedative premedication for surgical treatments [[94], [95], [96], [97]],anaesthesia induction [98], neonatal analgesia [99], postoperative anxiety [100,101], blood withdrawal anxiety [100], EEG premedication [[102], [103], [104]] and MRI premedication [105]. The results from these studies suggest that melatonin can be beneficial in the management of these disorders, or during these interventions.

### 6.3 Studies with prolonged-release melatonin in children

Three randomised controlled studies of controlled-release melatonin have been performed, comprising 220 children and adolescents age 2–17.5 years, all of whom had autism spectrum disorder or neurogenetic disorders [[106], [107], [108]]. Controlled-release melatonin improved sleep and caregivers' quality of life. Except for somnolence relevant adverse effects did not occur [108]. As studies comparing controlled-release melatonin with immediate-release melatonin in children have not yet been performed [78], the question remains unresolved as to which of these treatments is the best for children with circadian rhythm sleep disorders.

## 7 Safety of melatonin administration

To assess the safety of melatonin we reviewed the results of in vitro, animal, and human studies.

### 7.1 Animal and in vitro studies

Toxicity studies are summarized in Table 1. Single and repeated dose toxicity studies in rats and mice with melatonin given orally, intraperitoneally (i.p.), subcutaneously (s.c.) and intravenously (i.v.) at different doses only showed toxic effects at extremely high melatonin doses (>400 mg/kg). The LD50 values of i.p., s.c. and i.v. administration of melatonin were similar for mice and rats, but the LD50 for oral administration was lower in mice (1250 mg/kg) than rats (3200 mg/kg) [109]. For comparison, the daily recommended dose of 3–6 mg in a person weighing 70 kg equates to a value of 0.04–0.08 mg/kg, some tens of thousands of times less than the melatonin dose causing death in 50% of rats or mice.

**Table 1:** Summary of toxicity studies with melatonin. SD: single Dose. LT: long-term administration. GT: genotoxicity. CG: carcinogenicity. O: other toxicity studies. i.p.: intraperitoneally, s.c. subcutaneously, i.v. intravenously. dw: drinking water.

Study type	Animal	Route	Duration	Results	Reference
SD	Rats in vivo	Orally, i.p.,s.c., i.v.	Once-only	At doses >400 mg/kg: vasodilatation, piloerection, ptosis, impairment of righting reflex, lack of motor activity, decrease in body temperature and respiratory problems preceding death	[109]
LT	Aged rats and mice in vivo	10 mg/L in dw	16 months	Improved health and survival of aged rats and mice	[111,112]
LT	Diabetic mice and hypercholestaemia-susceptible rats in vivo	s.c or in dw		Enhanced survival	[114,115]
GT	In vitro non-mammalian cell system			No mutagenicity in bacterial strains.	[113,[116], [117], [118]]
GT	In vitro mammalian cell system			No chromosome aberrations and no clastogenic activity. Protective anti-clastogenic activity. Nop DNA strand breaks.	[113,117,119]
GT	In vivo cell system	5 mg/kg s.c.		Not mutagenic in mouse bone marrow cells; reduced chromosome aberration rates. Not mutagenic in mice sperm head anomaly test; reduced sperm head anomaly rates.	[120]
GT	In vivo cell system	10 mg/kg i.p.		No adverse effects on rat peripheral blood micronucleus test.	[121]
GT	In vivo study in rats or mice	4–10 mg/kg i.p.		Protective effects against genotoxic action of potassium dichromate, cobalt, ethanol, paraquat	[[122], [123], [124], [125]]
GT	In vitro study using human lymphocytes	0.2 mM		Anti-genotoxic effect on mercuric chloride and gossypol	[110,126]
CG	Transgenic mice at increased risk for prostate adenocarcinoma	10–20 mcg/L in dw	18 weeks	Strong prostate cancer inhibitory effect	[127]
CG	Transgenic mice susceptible for mammary tumours	50–200 mcg/kg per day via gavage	30 weeks	Reduced incidence and growth rate of mammary tumours	[128,144]
CG	Rats, mice susceptible for breast cancer	10–20 mcg/L in dw	>1 yr	No induction of uterine tumours, lower incidence of mammary tumours.	[129,130]

Long-term administration of melatonin [110] in drinking water (10 mg/L, resulting in a dose of approximately 1 mg/kg/day) improved the general health and survival of aged rats [111] or aged mice [112,113], diabetic mice [114] and hypercholesterolaemia-susceptible rats [115].

A full range of genotoxicity tests, including in vitro non-mammalian [113,[116], [117], [118]] and in vitro mammalian cell systems [113,117,119], and in vivo mammalian system [120,121] tests consistently found no evidence that melatonin is genotoxic (neither mutagenic or clastogenic). Furthermore, several studies in various in vitro/in vivo systems have reported the anti-genotoxic action of melatonin [[122], [123], [124], [125], [126]], usually ascribed to its antioxidant activity.

Medium term (18–30 week) and long term (>one year) studies showed that melatonin had a strong prostate cancer inhibitory effect [127], and decreased breast cancer tumour incidence and growth rate [[128], [129], [130]],in transgenic mice lines susceptible to breast cancer. Furthermore, melatonin did not induce uterine tumours in rats [131], and resulted in a lower incidence of mammary tumours in mice. The anti-cancer action of melatonin is thought to involve inhibition of cancer initiation, progression and metastasis [132], and results via a number of mechanisms, including antioxidant action, regulation of oestrogen metabolism, inhibition of telomerase activity, inhibition of metastasis, anti-angiogenesis, and activation of the immune system [133].

In diurnal primates, an oral melatonin dose of 0.005 mg/kg administered 2 h before lights-off time promoted significantly earlier sleep-onset. Long-term melatonin administration did not result in development of tolerance or sensitisation to melatonin effects on sleep [134].

Intravenous administration of melatonin (10 mg/kg) had no effect on blood pressure in the cat, and no effect on heart function in the dog [135]. Intraperitoneal administration of melatonin (8 mg/kg) had no effect on neurological behaviour in mice [136].

Rodent studies indicate that melatonin has a cardioprotective action in myocardial infarction. In a mouse model, melatonin pre-treatment (0.150 mg/kg i.p.) significantly reduced the infarct size [137]. Similar results were obtained in rats, with the cardioprotective effect thought to result from the free radical scavenging capacity of melatonin [138].

No treatment-related effects were found for the endocrine system, including serum concentrations of 17 beta-oestradiol, progesterone, prolactin, luteinising hormone, and the glandular area of mammary tissue as measured on day 20 of gestation, when doses of melatonin in the range 1–200 mg/kg/d by gavage were administered to groups of female rats on days 6–19 of gestation, while aversion to treatment (at doses> 100 mg/kg/d) and reduced maternal weight gain (at doses >150 mg/kg/d) were observed. There was no maternal morbidity/mortality, and foetuses showed normal development [139]. Oral administration of melatonin (4 mg/L in drinking water, ∼0.4 mg/kg/day) for 3 months in middle aged rats resulted in decreases in body weight, intra-abdominal adiposity, plasma leptin, and plasma insulin levels, whilst locomotor activity was restored to more youthful levels. Intraperitoneal administration of melatonin (4 mg/day for 4 months; the authors did not specify the weight of rats used) resulted in beneficial effects on blood cholesterol levels in rats genetically predisposed to development of hypercholesterolaemia [115].

Melatonin receptors are located on a wide range of cells throughout the body, not just the SCN [140]. Some of these may play a role on glucose metabolism [141]. Acute melatonin administration in humans impairs glucose tolerance in the morning and evening [142].

Since both melatonin and benzodiazepines bind to GABA receptors, there is potential for their interaction. Murine data obtained in vitro [143] and in vivo [136] support this concept, although it should be noted that very high doses (260 mg/kg) of melatonin were used in these studies.

#### 7.1.1 Animal studies on reproduction

The results of animal and in vitro studies as to the influence of melatonin on reproduction are summarized in Table 2. No adverse effects on fertilisation and early embryonic development were identified. Interpretation of such non-clinical data in relation to clinical use is complicated by the fact that species for which most data are available (rats, mice) are seasonal breeders ie a shorter photoperiod during winter suppresses sexual development/reproduction, whilst a longer photoperiod in spring suppresses melatonin synthesis and sexual development/reproduction begins. Since sexual maturation and the reproductive cycle in humans are not dependent on seasonal photoperiod, it is unlikely that melatonin plays a significant role in decreasing the development of sexual maturity [144].

**Table 2:** Summary of reproductive and developmental toxicity studies with melatonin. s.c.: subcutaneous. dw: drinking water.

	Dose	Period of administration	Results	Reference
Male Wistar rats	110 mcg s.c. Daily	Age 20–45 days	Reduced pituitary GnRH receptor content at 70 days of age	[150]
Pre-pubertal female Sprague–Dawley rats	110 mcg s.c. Daily	Age 20–70 days	Normal sexual maturation	[151]
Female Holzman rats	10 mcg/L in dw	Age 10–380 days	Delayed vaginal opening, no effect on oestrus cycle	[131]
Adult rats	4 mg/l in dw	12 weeks	No adverse effect on sexual behaviour	[152]
Sexually active male Wistar rats	10–100 mcg/kg intraperitoneally	Once before mating	No adverse effect on sexual behaviour	[153]
Embryonic in vitro studies in rat, mouse, and pig	10(-5) M to 10(-13) M for 48 or 72 h		No adverse effect on in vitro fertilisation and early embryonic development	[139,154,155]
Pregnant Sprague–Dawley rats	200 mcg/kg gavage	Gestational days 6–19	No toxic effect on embryo-foetal development	[139]
Pregnant rats	300 mcg/rat s.c.	Gestational days 8–21	No effect on litter size, live young birth weights and incidence of stillbirths	[155]

Several studies suggest that melatonin might improve progesterone function in human granulosa-lutein cells [145],follicular cells [146] and corpus luteum [147].Therefore melatonin has been suggested to be a relevant medication for improving ovarian and luteal function and in the early stages of pregnancy, opening new opportunities for the management of several ovarian-luteal and pregnancy diseases [148].

Melatonin doses are 10–100 fold higher than the level provided by a 3–6 mg dose in man. It should be noted that adults produce 20–40 mcg melatonin per night [149]. When a 15 kg child is administered 6 mg of melatonin, this is 150 times more than an adult produces each night.

### 7.2 Clinical studies in children

In the 19 randomised trials of exogenous melatonin for children with DSPD (n = 841) [77] no serious adverse effects were reported, irrespective of patient category or dose up to 10 mg/day; studies were typically of 4 weeks' duration, although some studies extended to 3 months. The safety of melatonin for children with DSPD-like features was confirmed in 4 meta-analyses [83,[156], [157], [158]]; in two of these analyses patients with neurodevelopmental disorders were included.

The long-term safety of melatonin supplementation in children was determined in 3 studies. One study included 44 children with neurodevelopmental disorders and treatment-resistant circadian rhythm sleep disorders [159]; the second comprised 94 children with ADHD and DSPD [160], and third study 51 children with DSPD [88]. The mean follow up intervals were 3.8, 3.7 and 3.1 years respectively. Participants of the latter study were additionally followed up to 10.8 years [89] without any substantial deviation of the development of children with respect to sleep quality, puberty development and mental health scores. In addition, the fourth study, including 95 children and adolescents with autism spectrum disorder (ASD) and neurogenetic disorders (NGD) with/without attention-deficit/hyperactivity disorder comorbidity, long-term treatment with 5 or 10 mg controlled-release melatonin over 39–52 weeks was both safe and efficacious [107].

Supplemental melatonin has been widely used in children for surgical premedication, and prior to EEG or magnetic resonance procedures. Furthermore melatonin is used for migraine prophylaxis, Dravet syndrome, atopic dermatitis, and to treat olanzapine-related weight gain in bipolar disorder. Safety outcomes of these 17 randomised trials including 1374 children are summarised in Table 3. Since an immediate-release melatonin formulation is required for such applications, and no commercially available immediate-release melatonin products licensed for use in children are available, the use of unlicensed products is necessitated; these can vary in quality from food grade supplements to off-license use of products licensed within the EC such as Bio-Melatonin.

**Table 3:** Summary of safety outcomes from randomised controlled studies of melatonin for indications other than DSPD in children.

Study	Indication	Number of subjects (n)/age range	Melatonin dose/formulation (as single dose unless otherwise indicated)	Reported adverse effects (vs control)
Fallah et al. [90]	Migraine prophylaxis	n = 80/5–15 yrs	0.3 mg/kg/day for 3 months	Daytime somnolence in 3 children
Myers et al. [91]	Dravet syndrome	n = 13/2–50 yrs	6 mg/day for 2 weeks	No adverse effects
Ardakani et al. [92]	Atopic dermatitis	n = 70/6–12 yrs	6 mg/day for 6 weeks	No adverse effects
Impellizzeri et al. [162]	Surgical premedication	n = 80/9–11 yrs	0.5 mg/kg to 20 mg max	No adverse effects
Mostafavi et al. [93]	Weight gain in bipolar disorder	n = 48/11–17 yrs	3 mg/day for 12 weeks	No adverse effects
Gitto et al. [94]	Surgical premedication	n = 92/5–14 yrs	0.5 mg/kg/day	No adverse effects
Marseglia et al. [100]	Blood withdrawal anxiety	n = 60/1–14 yrs	0.5 mg/kg/day to 5 mg max	No adverse effects
Fallah et al. [102]	EEG premedication	n = 60/1–8 yrs	0.3 mg/kg/day	No adverse effects
Almenrader et al. [98]	Anaesthesia induction	n = 87/12–71 yrs	0.3 mg/kg/day	No adverse effects
Gitto et al. [99]	Neonatal analgesia	n = 60/5–14 yrs	0.5 mg/kg/day	No adverse effects
Sander et al. [103]	EEG premedication	n = 50/1–18 yrs	3 mg < 15 kg 6 mg > 15 kg	No adverse effects
Ozcengiz et al. [101]	Post-operative anxiety	n = 100/3–9 yrs	0.1 mg/kg/day	No adverse effects
Kain et al. [95]	Surgical premedication	148/2–8 yrs	0.05–0.4 mg/kg/day	No adverse effects
Isik et al. [96]	Surgical premedication	n = 60/508 yrs	3 mg/day	No adverse effects
Sury [105]	Sedation for magnetic resonance imaging	n = 98/0.3–4 yrs	3–6 mg/day	No adverse effects
Samarkandi et al. [97]	Surgical premedication	n = 105/2–5 yrs	0.1–0.5 mg/kg/day	No adverse effects
Wassmer et al. [104]	EEG sleep study	n = 163/1–16 yrs	2–10 mg/day	No adverse effects

Melatonin influences the human gonadotrophin-releasing hormone pulse generator, suggesting that melatonin could be used as contraceptive [161]. However, we could find no published studies of contraceptive effects in humans.

### 7.3 Adverse events

Severe adverse events associated with oral melatonin are scarce [[163], [164], [165]]. Mild generalized epilepsy was mentioned in two studies [84,166]. However, melatonin also has an anticonvulsant action as shown by animal and human studies [167]. Nevertheless, a Cochrane review could not draw any conclusion about the role of melatonin in reducing seizure frequency or improving quality of life in people with epilepsy [168,169]. Hypothermia was reported in a child with autism after melatonin ingestion, suggested to be an extreme variant of the normal physiologic action of melatonin on decreasing body temperature [169]. In one case, melatonin was suspected to be involved in the sudden death of a twin infant [170]. A critical systematic review of clinical evidence for adverse events associated with oral administration concluded that melatonin supplementation in humans has a generally favourable safety profile with some exceptions, relating to fatigue, mood or psychomotor and neurocognitive performance. These events were generally minor, short-lived and easily managed, eg by dosing in accordance with natural circadian rhythms [163]. Also in the authors' clinical experience, sometimes mentioned vivid dreams and frequent awakenings at night are usually short lived (clinical experience, MS). Sleep maintenance problems may point at a too high dose of melatonin (clinical experience, MS).

Finally, post-marketing data for an immediate-release melatonin formulation (Bio-Melatonin), used in the UK since 2008 as an unlicensed medicine for sleep disturbance in children, recorded no adverse events to date on sales of approximately 600,000 packs, equivalent to some 35 million individual 3 mg tablet doses (MHRA yellow card adverse event reporting scheme, and personal communication, DM).

### 7.4 Overall conclusion on safety

Melatonin has low acute toxicity, with LD50 values some 4 orders of magnitude greater than melatonin doses recommended in humans. Long-term administration of melatonin in rodents resulted in improved general health, and survival of aged animals. Melatonin is not genotoxic or carcinogenic. Sexual maturation in young rats was somewhat delayed, but not prevented following melatonin administration in doses 100-fold greater than the proposed dose in humans. Interpreting non-clinical data in relation to clinical use is complicated by the fact that species for which most data are available (rats, mice) are seasonal breeders ie a shorter photoperiod during winter suppresses sexual development/reproduction, whilst a longer photoperiod in spring suppresses melatonin synthesis and sexual development/reproduction begins. In animals with a reproduction cycle of once per year, this phenomenon is even more pronounced. For sheep, melatonin implant injections are authorized in Europe to stimulate twice yearly breeding, overruling their seasonality. As humans are non-seasonal breeders, this is not applicable in man. Thus caution should be exercised in the extrapolation of non-clinical or veterinary data to man.

No adverse effects of melatonin on early embryonic development in rodents were detected in vitro or in vivo. Similarly administration of melatonin to pregnant rats had no toxic effect on embryo-foetal development, and the No Observed Adverse Effect Level value established of 200 mg/kg/day is approximately one thousand fold greater the equivalent recommended dose in man. Limited indications of mild maternal toxicity were obtained in pregnant mice or rats treated with very high doses of melatonin-the maternal Lowest Observed Adverse Effect value was 200 mg/kg/day.

Melatonin administration in the morning decreased glucose tolerance primarily by decreasing insulin release, and in the evening, by decreasing insulin sensitivity. The clinical significance for patients with impaired glucose tolerance of this feature of chrononutrition [171] found in a study with healthy humans [142], has not yet been studied. Nevertheless, when patients with impaired glucose tolerance start melatonin treatment at night it could be useful to ask them for signs and symptoms of increased glucose levels. Eventually mealtimes could then be advanced [142].

## 8 Discussion

Clinical efficacy studies have demonstrated that melatonin is remarkably effective for children and adolescents with sleep problems, provided that these sleep problems are due to DSPD, and that melatonin is administered at the correct time and in the correct dose. Furthermore, animal, in vitro and human safety studies did not reveal any evidence of potentially harmful effects of melatonin treatment in children. We did not find indications for clinical relevant effects on cardiovascular, immune, and metabolic systems.

Sleep problems due to DSPD may resemble those of insomnia. For example, delayed sleep-onset is one of the main characteristics of DSPD, but it also one of the features of insomnia, since insomnia is defined as difficulty in initiating/maintaining sleep or nonrestorative sleep. Delayed sleep-onset in DSPD is a consequence of delayed circadian rhythmicity, which consequently usually responds to melatonin treatment. However, sleep-onset insomnia can also be due to insufficient sleep hygiene, or insomnia. The first choice of treatment for insomnia is cognitive behaviour therapy (CBT-I) [172]. Usually melatonin treatment is not helpful then. Furthermore, when the sleep–wake rhythm is delayed for a long time due to DSPD, and lifestyle factors are not in accordance with the biological clock, sleep maintenance problems may arise in addition to sleep-onset and sleep offset issues according to our clinical experience. These sleep problems can also be diagnosed as insomnia; however, not CBT-I, but melatonin treatment will then be helpful. Consequently, for optimal treatment with melatonin, a correct diagnosis is crucial. When the sleep–wake rhythm alone is insufficient to diagnose DSPD, assessment of the DLMO can help considerably [48]. However, clinicians should be aware of a possible pitfall: according to our experience DLMO fluctuates for the first few weeks after stopping melatonin [160]. Therefore we advise measurement of DLMO no earlier than 4–6 weeks after stopping melatonin treatment (clinical experience, MS).

Ideally the DLMO should be known, not only for aiding diagnosis, but also for optimal melatonin treatment. The melatonin concentration is readily determined in saliva collected at the children's home, and several laboratories are able to establish the DLMO, including saliva samples sent from abroad (eg www.melatoninecheck.nl). Nevertheless, practical drawbacks may make it unfeasible to determine DLMO. In that case, we would advise starting melatonin treatment 3–5 h before bedtime. Try to find the lowest effective melatonin dose, starting with 1 mg in children between 6 and 18 years. If after one week no change occurs, increase the dose by 1 mg weekly until an effect occurs. When a 1 mg melatonin dose is already effective, try to lower the dose until a minimal effective dose is reached. If there is no effect using a 3–6 mg dose, stop melatonin treatment and try to measure DLMO, or reconsider the diagnosis [48] (clinical experience, MS).

The present review did not reveal evidence of potentially harmful effects of melatonin treatment in children. Concerns for possible adverse effects of melatonin on reproductive function are not justified on the basis of evidence reviewed in the present article. In vitro and animal studies indicate that melatonin can be used safely in the recommended doses. This is supported by the critical systematic review of adverse events associated with oral administration of melatonin [168], and a search of Medline for adverse effects of melatonin, identifying 789 papers limited to ‘human’ and 543 to ‘animal’ [6]. Nevertheless melatonin has the potential to cause problems if it is used incorrectly. Thus when melatonin is administered at the wrong time (usually shortly before bedtime without knowing DLMO), or in the wrong dose (usually too high), successful treatment of the sleep disorder may be postponed unnecessarily. During our 25-years' experience we saw some medically important side effects including diarrhoea, headache, and enuresis, which have not received much attention in the literature. When diarrhoea is caused by melatonin, we suggest treatment should be stopped, as we have found all conventional methods to stop this intestinal problem to be unsuccessful. The same scenario applies to melatonin induced persistent headache or enuresis (clinical experience, MS). Other treatments should then be considered ie bright light, behavioural interventions or adaptation of lifestyle to the delayed biological clock [58].

A rapidly increasing number of children are using melatonin, with the numbers doubling from 2004 to 2011 [173]. The Dutch Generation R study reported in 2019 that 6% of school-aged children used melatonin regularly. Caregiver- and child-reported shorter total sleep time appeared to be the indication for melatonin use, without taking into account the cause of the poor sleep [8].

To conclude, melatonin is a safe chronobiotic drug for the treatment of delayed sleep–wake phase disorder in children, at least in the short term, provided that it is administered at the right time and in the right dose. Treatment should be tailored to the specific patient in relation to the particular status of the biological clock. As this status may change through the years we recommend stopping melatonin treatment at least once a year (preferably during the summer holidays).

## 9 Keypoints

• Clinical efficacy studies demonstrated that melatonin is remarkably effective for children and adolescents with sleep problems, provided that these sleep problems are due to DSPD, and that melatonin is administered at the correct time and in the correct dose.
• Animal, in vitro and human safety studies did not reveal any evidence of potentially harmful effects of melatonin treatment in children.

## CRediT author statement

D. Mantle: conceptualization, methodology, investigation, review and editing, visualization. M. Smits: writing original draft, investigation, review and editing, project administration. M. Boss: methodology, visualization, review and editing. I. Miedema: validation, review and editing. I. van Geijlswijk: supervision, validation, review and editing.
